# Residential self-selection bias in the estimation of built environment effects on physical activity between adolescence and young adulthood

**DOI:** 10.1186/1479-5868-7-70

**Published:** 2010-10-04

**Authors:** Janne Boone-Heinonen, David K Guilkey, Kelly R Evenson, Penny Gordon-Larsen

**Affiliations:** 1 Department of Nutrition, University of North Carolina at Chapel Hill, Carolina Population Center, 123 West Franklin St., Campus Box 8120, Chapel Hill, 27516-3997, USA; 2 Department of Economics, University of North Carolina at Chapel Hill, Carolina Population Center, 123 West Franklin St., Campus Box 8120, Chapel Hill, 27516-3997, USA; 3 Department of Epidemiology, University of North Carolina at Chapel Hill, Bank of America Center, 137 East Franklin Street, Suite 306, Campus Box 8050, Chapel Hill, 27514, USA

## Abstract

**Background:**

Built environment research is dominated by cross-sectional designs, which are particularly vulnerable to residential self-selection bias resulting from health-related attitudes, neighborhood preferences, or other unmeasured characteristics related to both neighborhood choice and health-related outcomes.

**Methods:**

We used cohort data from the National Longitudinal Study of Adolescent Health (United States; Wave I, 1994-95; Wave III, 2001-02; n = 12,701) and a time-varying geographic information system. Longitudinal relationships between moderate to vigorous physical activity (MVPA) bouts and built and socioeconomic environment measures (landcover diversity, pay and public physical activity facilities per 10,000 population, street connectivity, median household income, and crime rate) from adolescence to young adulthood were estimated using random effects models (biased by unmeasured confounders) and fixed effects models (within-person estimator, which adjusts for unmeasured confounders that are stable over time).

**Results:**

Random effects models yielded null associations except for negative crime-MVPA associations [coefficient (95% CI): -0.056 (-0.083, -0.029) in males, -0.061 (-0.090, -0.033) in females]. After controlling for measured and time invariant unmeasured characteristics using within-person estimators, MVPA was higher with greater physical activity pay facilities in males [coefficient (95% CI): 0.024 (0.006, 0.042)], and lower with higher crime rates in males [coefficient (95% CI): -0.107 (-0.140, -0.075)] and females [coefficient (95% CI): -0.046 (-0.083, -0.009)]. Other associations were null or in the counter-intuitive direction.

**Conclusions:**

Comparison of within-person estimates to estimates unadjusted for unmeasured characteristics suggest that residential self-selection can bias associations toward the null, as opposed to its typical characterization as a positive confounder. Differential environment-MVPA associations by residential relocation suggest that studies examining changes following residential relocation may be vulnerable to selection bias. The authors discuss complexities of adjusting for residential self-selection and residential relocation, particularly during the adolescent to young adult transition.

## Background

Built environment characteristics such as walkability [1,2] and availability of recreation centers [3,4] are associated with physical activity (PA) in a growing literature. However, existing research is dominated by cross-sectional studies, which are particularly vulnerable to residential self-selection bias resulting from unmeasured neighborhood selection factors related to built environment exposures and PA [5,6]. Neighborhood selection factors may include preference for PA resources, which could affect neighborhood choice and PA level. Similarly, social and financial resources not only influence where individuals can afford to live but also shape perceived barriers to PA. Furthermore, traditional covariate adjustment cannot adequately control for neighborhood preferences and other residential selection factors that are difficult or impossible to measure.

Longitudinal designs can address residential self-selection bias by establishing temporality and controlling for unmeasured characteristics. In two key longitudinal studies [7,8], investigators used "first difference" models to estimate the influence of urban form on travel behavior or obesity. First difference models and a similar method, "fixed effects" models, use within-person estimators to control for unmeasured characteristics that remain constant throughout the study period [6,9] (e.g., genetics or resilient attitudes toward exercise) by analyzing variation in the exposure and outcome within person, over time. Within-person estimation is especially valuable when confounders are difficult to measure (e.g., residential selection factors), and is most appropriate for exposure-outcome relationships with short lag times [10] (e.g., theorized built environment influences on PA). Recent longitudinal studies [11-14] investigating built environment effects on PA do not use within-person estimation to control for unmeasured characteristics.

Furthermore, the few relevant existing studies which use within-person estimation [2,7,8,15] examine changes in behavior or body weight related to changes in urban form resulting from residential relocation. However, the environment can change around stationary residents. Furthermore, residential relocation is often triggered by events such as marriage or employment changes [16], which may also influence health-related behaviors. Therefore, restricting to those who move residences may induce selection bias [17].

Our primary objective was to estimate within-person effects of time-varying, objectively measured built and socioeconomic environment characteristics on moderate to vigorous PA (MVPA) in a nationally representative sample. Secondary objectives were to (a) assess the influence of time invariant, unmeasured characteristics on environment-PA associations by comparing within-person estimates to naïve estimates which do not address unmeasured characteristics, and (b) explore selectivity related to residential relocation. This paper reports the results of these objectives, followed by a discussion of the complexities of adjusting for residential self-selection and residential relocation, particularly during the adolescent to young adult transition.

## Methods

### Study population and data sources

We used Wave I (1994-95) and III (2001-02) data from The National Longitudinal Study of Adolescent Health (Add Health), a cohort study of 20,745 adolescents representative of the U.S. school-based population in grades 7 to 12 (11-22 years of age) in 1994-95 followed into adulthood (18-26 years at Wave III). Add Health included a core sample plus subsamples of selected minority and other groupings collected under protocols approved by the Institutional Review Board at the University of North Carolina at Chapel Hill. The survey design and sampling frame have been discussed elsewhere [18].

Using a geographic information system (GIS), we linked respondents' Wave I and III residential locations to community-level data theorized to influence obesity and obesity-related behaviors. Among respondents in the probability sample (n_Wave I _= 18,924, n_Wave III _= 14,322), residential locations were determined from geocoded home addresses with street-segment matches (n_Wave I _= 15,480, n_Wave III _= 12,263), global positioning system (GPS) measurements (n_Wave I _= 2,966, n_Wave III _= 1,148), ZIP/ZIP+4/ZIP+2 centroid match (n_Wave I _= 205, n_Wave III _= 647) and geocoded school location (n_Wave I _= 243; not applicable in Wave III, n = 264 unmatched). Comparison of individual-level and environmental measures across location sources suggest that respondent locations identified with GPS or ZIP codes (compared to geocoded addresses) were located in rural areas. Such differences were expected because rural residents more often use Post Office Boxes or other addresses that cannot be geocoded; that is, multiple location sources allowed us to include such respondents, thereby minimizing selection bias. Residential locations were linked to attributes of circular areas of various radii surrounding each wave-specific respondent residence (Euclidean neighborhood buffer) and block group, tract, and county attributes from time-matched U.S. Census and other data (see Study variables, below), which were merged with individual-level Add Health interview responses.

Of 18,924 Wave I respondents in the probability sample, 6% refused participation and 19% could not be located or were unable to participate for other reasons, leaving 14,322 Wave III respondents. Exclusions included mobility disability (n = 87) or self-reported pregnancy at Wave I or III (n = 578) and Native Americans due to small sample size (n = 121). Of the remaining sample (n = 13,546), those missing individual-level variables (n = 266), environmental variables (n = 568), or both (n = 11) were excluded. Those excluded due to missing data (n = 845) were generally similar to the analytical sample (n = 12,701) with regard to Wave I and III individual sociodemographics, MVPA, and environmental variables. Exceptions included lower census tract-level median income and Wave III landscape diversity, and higher Wave III MVPA in excluded respondents (data not shown).

### Study variables

#### GIS-derived environmental characteristics

##### Geographic Units

We used neighborhood buffer sizes (e.g., 1 or 3 k) based on research showing that MVPA was most strongly and consistently associated with street connectivity within smaller areas (1 k) and with PA facilities within larger areas (3 k) [19], consistent with theorized higher incentive to travel to PA facilities and engagement in street-based activities closer to home. We selected census tracts for census variables based on similar sensitivity analysis (unpublished data), while crime data were available only at the county level.

##### Built and socioeconomic environment measures

We selected built and socioeconomic environment measures shown to adequately represent multidimensional environmental constructs [20]. Table 1 presents variable descriptions, data sources, and geographic unit. Briefly, ***pay ***and ***public PA facilities counts ***were obtained from Dun and Bradstreet, a dataset of U.S. businesses validated against a field-based census [21]. We then calculated PA facility availability (counts per 10,000 population). In contrast with raw counts or distance to facilities, such population-scaled measures may help to separate availability of facilities from density of development, which are independently related to behavior [20,22].

**Table 1:** Built and socioeconomic environment source measures: data sources and variable descriptions^1^

		Data Year			
Measure	Data Source	Wave I	Wave III	Geographic Area^2^	Variable description
Street connectivity (Alpha index)	ESRI StreetMap	1999^3^	2003^3^	1 k	Ratio of observed to maximum possible route alternatives between nodes (intersections); high values indicate high street connectivity.
Pay facilities availability (count per 10,000 population)	Dun and Bradstreet	1995	2001	3 k	Number of pay facilities per 10,000 population (population count from Census, see below). Includes Instruction (e.g., dance studios, basketball instruction, martial arts), Member (e.g., athletic club and gymnasium, tennis club, basketball club), and Public fee (e.g., physical fitness facilities, bicycle rental, public golf courses) facilities identified by 8-digit Standard Industrial Code.
Public facilities availability (count per 10,000 population)	Dun and Bradstreet	1995	2001	3 k	Number of public facilities per 10,000 population (population count from Census, see below). e.g., public beach, pools, tennis courts, recreation centers identified by 8-digit Standard Industrial Code.
Landscape diversity (Simpson's diversity index)	National land cover dataset	1992	2001	1 k	Represents the probability that any two pixels selected at random are different patch types.
Population count	U.S. Census	1990	2000	3 k	Count of persons within buffer, calculated by averaging census block-group population counts, weighted according to the proportion of block-group area captured within 3 k
Median household income	U.S. Census	1990	2000	CT	Median household income. Wave I values were inflated to 2000 dollars using the Consumer Price Index.
Crime rate	Uniform Crime Reporting data	1995	2001	Co	Number of non-violent and violent crimes per 100,000 population (provided in source dataset; buffer-based population counts listed above were not used to calculate crime rate)

^1^From the National Longitudinal Study of Adolescent Health (United States; Wave I, 1994-95; Wave III, 2001-02), Obesity Neighborhood Environment Database (ONEdata). Measures selected based on prior research showing them to adequately represent multidimensional environmental constructs

^2^Selected neighborhood definitions were selected because they yielded the strongest associations between environment measures and physical activity in previous analysis [20].

^3 ^Wave I used ESRI Streetmap 2000 (reflecting ground conditions in 1999), Wave III used ESRI Streetmap Pro (reflecting ground conditions in July 2003)

1 k, 3 k, 1 and 3 kilometer Euclidean buffer; CT, census tract; Co, County; U.S., United States

Simpson's Diversity Index, an indicator of ***landscape diversity ***and complexity [23], was calculated using Fragstats software [24]. ***Alpha index ***indicated the degree of street connectivity [25], which provides numerous, often more direct route options [26]. Socioeconomic environment measures included census tract-level ***median household income ***and county-level ***non-violent and violent crime rate ***per 100,000 population.

To account for slight inaccuracies in geocoded locations and inconsequential moves, ***residential relocation ***(mover vs. non-movers) was defined as > 1/4 mile Euclidean distance between Wave I and III residential locations.

#### Individual-level variables

Weekly frequency (bouts) of leisure-time MVPA (skating & cycling, exercise, and active sports) was ascertained at Waves I and III using a standard, interview administered activity recall based on questionnaires validated in other epidemiologic studies [27,28]. The questionnaire included activities relevant to adolescents (11-22 years) at Wave I and was modified at Wave III (18-26 years) to include age-appropriate activities, so Wave III bouts were scaled for comparability with Wave I [29]. Semi-continuous MVPA was rounded to the nearest integer for appropriate modeling as a count variable.

Individual-level sociodemographic control variables included Wave I self-identified race (white, black, Asian, Hispanic), parent-reported annual household income and highest education attained (< high school, high school or GED, some college, ≥ college degree), and age at Wave I and III interviews. To account for regional differences in MVPA and neighborhood environments, we controlled for administratively determined U.S. region (West, Midwest, South, Northeast). Socioeconomic position in young adulthood involves a complex array of behaviors and achievements [30,31] which are potential predictors of residential relocation, so we used parent income and education to indicate socioeconomic position in both waves.

### Statistical analysis

#### Descriptive analysis

Individual-level and environment variables were compared by residential relocation status using adjusted Wald tests and design-based F-tests (95% confidence level) for continuous and categorical variables, respectively. Analyses were weighted for national representation and corrected for complex survey design using Stata 10.1 survey commands. To address skewness, we report median and interquartile range and performed statistical tests on natural-log transformed pay and public facility availability and median household income.

#### Regression analysis

Within-person effects of environment measures on MVPA bouts from adolescence (Wave I) to young adulthood (Wave III) were estimated using fixed effects Poisson regression (Objective 1). Fixed effects (versus first differences) accommodate our nonlinear dependent variable. By analyzing deviations of the outcome and exposures from person-specific means, fixed effects models remove person-specific error and are therefore not biased by time invariant unmeasured characteristics. As demonstrated elsewhere [6,8,32] and in additional file 1, appendix A, interpretation of the coefficients is unchanged from traditional regression models. In contrast, "random effects" estimates incorporate both between- and within-person variation and thus do not control for unmeasured characteristics that vary or remain constant over time (naïve estimation; Objective 2a) [33].

The Hausman specification test formally compared fixed and random effects estimates. All models were fit using the Stata 10.1 xtpoisson function [34], which provided comparable estimates but does not accommodate probability weights. Sample weighted, school cluster-corrected, within-person estimates obtained using an alternative method [32] were substantively similar, but comparable random effects estimates were not possible given the available software. Random effects models corrected for school-level clustering by including school indicator variables [35]; higher-level clustering is subsumed into between-person variation which does not influence fixed effects regression models.

The MVPA distribution was overdispersed (the standard deviation was larger than assumed by the Poisson distribution), but the conditional likelihood for the negative binomial distribution required for fixed effects models is problematic [32]. However, additional error terms in random and fixed effects models [36] and correction for school-level variation may help to address overdispersion by allowing for sources of variability not included in a standard Poisson model. Estimates from cross-sectional Poisson and negative binomial models are virtually identical.

Buffer-based environment measures were individual-level variables. While census tracts or counties could comprise a third level in multilevel analysis, they are not nested within schools, our primary sampling unit and more important source of clustering. Additionally, our data were sparse (average 8 and 2.3 respondents per census tract in Wave I and III, respectively) and unbalanced (1-275 and 1-95 respondents per census tract in Wave I and III, respectively), so multilevel analysis may have produced biased estimates [37]. Intraclass correlations for ln(MVPA) were minimal (0.03 in both Waves; ICC's are not definable for Poisson distributed outcomes).

Natural log transformations of environment measures linearized relationships with MVPA bouts in preliminary analysis. Because both the dependent and independent variables were logged, model coefficients were interpreted as elasticities, or the percent change in MVPA bouts predicted from a 1% change in the independent variable. Time invariant individual-level variables were included in random effects models but are not estimated in fixed effects models. Time varying age was included in both models. Sex interactions with each environmental variable were tested; for comparability, interaction terms were retained if significant (Wald p < 0.10) in the random or fixed effects model. Further interaction with residential relocation status (Objective 2b) in fixed effects models was examined by including significant (Wald p < 0.10; lower order terms were retained) two- and three-way interactions between residential relocation status, sex, and each environment measure. When one or more interactions were included in the model, group-specific associations were reported.

## Results

Individual-level characteristics are presented in Table 2. 68.5% (SE 1.2%) of the analytical sample moved between Waves I and III (data not shown), and changes in environmental measures observed between Waves I and III (Table 3) provided sufficient variability for estimation of within-person effects, even for non-movers.

**Table 2:** Sociodemographic characteristics in adolescence (Wave I, 1994-95) and young adulthood (Wave III, 2001-02) [mean/% (SE)]^1^

	Male				Female			
		
	Total (n = 6,242)	Movers^2 ^(n = 4,065)	Non-movers (n = 2,177)	*P*^3^	Total (n = 6,459)	Movers^2 ^(n = 4,460)	Non-movers (n = 1,999)	*P*^3^
MVPA - Wave I (mean, bouts/week)	7.2 (0.1)	7.5 (0.2)	7.1 (0.1)	0.05	5.7 (0.1)	5.9 (0.1)	5.6 (0.1)	0.03
MVPA - Wave III (mean, bouts/week)	3.2 (0.1)	3.4 (0.1)	3.2 (0.1)	0.10	2.5 (0.1)	2.6 (0.1)	2.4 (0.1)	0.02
Age - Wave I (mean)	15.5 (0.1)	15.7 (0.1)	15.2 (0.1)	< 0.001	15.3 (0.1)	15.4 (0.1)	14.9 (0.1)	< 0.001
Age - Wave III (mean)	21.9 (0.1)	22.1 (0.1)	21.6 (0.1)	< 0.001	21.7 (0.1)	21.8 (0.1)	21.3 (0.2)	< 0.001
Parental household income - Wave I (mean, in 10,000's U.S. dollars)	43.1 (1.5)	43.2 (1.7)	43.0 (1.4)	0.9	44.6 (1.6)	44.0 (1.7)	46.1 (1.8)	0.07
Race/ethnicity (%)				< 0.001				0.01
White	68.4 (2.9)	71.1 (2.8)	62.8 (3.7)		69.9 (2.9)	71.7 (2.8)	65.8 (3.7)	
Black	15.5 (2.1)	15.4 (2.1)	15.8 (2.4)		14.9 (2.0)	14.8 (2.1)	15.0 (2.2)	
Asian	3.7 (0.7)	2.9 (0.6)	5.1 (1.2)		3.3 (0.7)	3.0 (0.6)	4.1 (1.1)	
Hispanic	12.4 (1.8)	10.6 (1.5)	16.2 (2.7)		11.9 (1.8)	10.6 (1.6)	15.0 (2.8)	
Highest parental education (%)				0.09				0.8
< High school	14.7 (1.4)	13.6 (1.3)	16.9 (1.9)		15.2 (1.4)	15.4 (1.4)	14.9 (1.8)	
High school/GED	31.4 (1.3)	31.6 (1.5)	31.0 (1.6)		32.3 (1.3)	31.8 (1.4)	33.6 (1.6)	
Some college	28.6 (1.0)	28.6 (1.1)	28.4 (1.4)		27.0 (0.9)	27.2 (1.1)	26.6 (1.4)	
College or greater	25.3 (1.7)	26.1 (1.9)	23.7 (1.9)		25.5 (1.7)	25.7 (1.8)	24.9 (1.9)	
Region (%)				0.01				0.001
West	15.6 (1.4)	15.4 (1.6)	16.0 (2.0)		16.4 (1.4)	15.7 (1.5)	18.2 (2.3)	
Midwest	30.4 (2.3)	32.2 (2.8)	26.7 (2.2)		32.6 (2.6)	33.9 (2.9)	29.6 (3.0)	
South	39.4 (1.8)	40.0 (2.1)	38.1 (2.5)		36.5 (1.8)	38.5 (2.2)	32.0 (2.4)	
Northeast	14.6 (0.9)	12.4 (1.1)	19.2 (1.9)		14.4 (1.0)	11.9 (1.3)	20.3 (2.1)	

^1^National Longitudinal Study of Adolescent Health (United States)

^2^Residential relocation defined as > 1/4 mile Euclidean distance between Wave I and Wave III residential locations

^3^2-sided test of difference between movers and non-movers in males and females determined from adjusted Wald tests (continuous variables) and design-based F-tests (categorical variables), weighted and corrected for clustering.

GED, Graduate Equivalency Degree; MVPA, moderate-vigorous physical activity; SE, standard error

**Table 3:** Baseline and changes in built and socioeconomic environment characteristics between adolescence (Wave I, 1994-95) and young adulthood (Wave III, 2001-02), by residential relocation status^1 ^

	Movers (n = 8,525)		Non-movers (n = 4,176)		
			
Measure (geographic area^2^)	mean (SE)	median (IQR)	mean (SE)	median (IQR)	*P*^3^
Landscape diversity (1 k)					
Baseline	0.53 (0.01)	0.58 (0.43, 0.67)	0.54 (0.01)	0.58 (0.46, 0.67)	0.3
Change (Wave III-Wave I)	-0.01 (0.01)	-0.02 (-0.15, 0.12)	-0.04 (0.01)	-0.03 (-0.14, 0.06)	0.002
Pay facility availability (count/10,000 population) (3 k)					
Baseline	2.64 (0.23)	1.71 (0.00, 3.71)	2.43 (0.20)	1.61 (0.34, 3.40)	1.0
Change (Wave III-Wave I)	2.00 (0.21)	1.38 (-0.05, 3.98)	2.10 (0.23)	1.02 (0.06, 3.04)	0.7
Public facility availability (count/10,000 population) (3 k)					
Baseline	0.30 (0.05)	0.00 (0.00, 0.29)	0.28 (0.05)	0.00 (0.00, 0.31)	0.7
Change (Wave III-Wave I)	0.33 (0.05)	0.00 (0.00, 0.54)	0.18 (0.05)	0.00 (0.00, 0.30)	0.02
Alpha street connectivity (1 k)					
Baseline	0.31 (0.02)	0.30 (0.22, 0.38)	0.33 (0.02)	0.30 (0.22, 0.38)	0.5
Change (Wave III-Wave I)	-0.002 (0.019)	-0.005 (-0.097, 0.077)	-0.018 (0.016)	-0.003 (-0.023, 0.012)	0.5
Median household income, 1,000's U.S. dollars (CT) ^4^					
Baseline	38.9 (1.3)	37.3 (27.5, 46.7)	41.2 (1.3)	39.8 (28.7, 51.5)	0.002
Change (Wave III-Wave I)	0.3 (1.0)	1.9 (-7.8, 9.8)	2.4 (0.3)	2.5 (-1.6, 5.8)	0.02
Crime, per 100,000 population (Co)					
Baseline	5,300 (247)	5,369 (3,072, 6,975)	5,547 (238)	5,528 (3,647, 6,459)	0.05
Change (Wave III-Wave I)	-551 (171)	-669 (-1,950, 309)	-879 (161)	-1,081 (-1,645, -350)	0.005

^1 ^National Longitudinal Study of Adolescent Health (United States). Residential relocation defined as > 1/4 mile Euclidean distance between Wave I and Wave III residential locations

^2 ^Geographic areas consistent with the strongest associations with MVPA in a previous study were selected for each variable.

^3^2-sided test of difference between movers and non-movers in males and females determined from adjusted Wald tests (continuous variables) and design-based F-tests (categorical variables), weighted and corrected for clustering. Statistical tests were performed on natural log-transformed pay facilities, public facilities, and median household income to correct for skewness.

^4 ^Wave I values inflated to 2000 U.S. dollars

1 k and 3 k, radius of Euclidean neighborhood buffer in kilometers (k); CT, Census Tract; Co, County; IQR, Interquartile Range; SE, standard error

Within-person estimates indicated that with 1% greater pay facilities in the neighborhood, MVPA bouts were 0.024% higher in males; corresponding associations were negative but not significant in females (Table 4). MVPA was negatively associated with crime and, for females in fixed effects models, marginally with median household income. Landscape diversity, public facility availability, and alpha index were unrelated to MVPA.

**Table 4:** Random and within-person effect estimates of built and socioeconomic environment characteristics on MVPA between adolescence (Wave I, 1994-95) and young adulthood (Wave III, 2001-02)^1^

	Random Effects		Within-person Effects	
	Elasticity	95% CI	Elasticity	95% CI
Landscape diversity	-0.008	(-0.024, 0.008)	-0.018	(-0.037, 0.001)
Pay facility availability (count/10,000 population)				
Males	0.014	(0.000, 0.029)	0.024*	(0.006, 0.042)
Females	-0.012^2^	(-0.027, 0.004)	-0.016^2^	(-0.035, 0.004)
Public facility availability (count/10,000 population)	0.008	(-0.016, 0.032)	0.002	(-0.025, 0.030)
Alpha Index	-0.015	(-0.088, 0.058)	-0.002	(-0.097, 0.092)
Median household income (U.S. dollars)				
Males	-0.019	(-0.048, 0.010)	0.022	(-0.016, 0.060)
Females	0.014^2^	(-0.016, 0.044)	-0.039	(-0.077, 0.000)
Crime (per 100,000 population)				
Males	-0.056*	(-0.083, -0.029)	-0.107*	(-0.140, -0.075)
Females	-0.061*	(-0.090, -0.033)	-0.046^2^	(-0.083, -0.009)

^1 ^National Longitudinal Study of Adolescent Health (United States; n = 12,701). Estimated from Poisson random and fixed effects regression modeling MVPA as a function of six natural log-transformed built and socioeconomic environment measures. Fixed effects models adjusted for time varying age and do not estimate parameters for time invariant individual-level variables; random effects models additionally adjusted for time invariant sex, race, parental income and education, and region.

^2^Statistically significant (p < 0.1) interaction with sex; sex interactions were included if significant in either random or fixed effects models.

*Statistically significant elasticity (2-sided p < 0.05)

CI, Confidence Interval; MVPA, moderate-vigorous physical activity (bouts per week); U.S., United States

The Hausman specification test rejected the null hypotheses (p < 0.001) that there is no correlation between unexplained person-specific variation and the independent variables. That is, changes in estimates after controlling for time invariant, unmeasured characteristics by applying the within-person estimator were statistically significant. Compared to random effect estimates, within-person elasticities were larger for pay facility availability and, in males, almost two times larger for crime rate. In females, the within-person estimator attenuated negative random effects estimates for crime and reversed the association to the counter-intuitive direction (marginally significant) for median household income (Table 4).

Several associations varied by residential relocation status and sex (Table 5). Elasticities between MVPA bouts and crime were substantially larger in non-movers than movers, and landscape diversity was negatively associated with MVPA only in non-movers. Public facility availability was positively associated with MVPA in female movers only, with variation in magnitude and direction by sex- and relocation status. Model coefficients and p-values corresponding to Tables 4 and 5 are reported in additional file 2, appendix B.

**Table 5:** Variation in within-person effect estimates of built and socioeconomic environment characteristics on MVPA between adolescence (Wave I, 1994-95) and young adulthood (Wave III, 2001-02) by residential relocation status^1^

	Movers^2 ^		Non-movers^2^		Total sample^2^	
	Elasticity	95% CI	Elasticity	95% CI	Elasticity	95% CI
Landscape diversity	-0.004	(-0.026, 0.017)	-0.072*	(-0.116, -0.029)	--^2^	
Pay facility availability (count/10,000 population)						
Males	--^2^		--^2^		0.027*	(0.009, 0.045)
Females	--^2^		--^2^		-0.022*	(-0.042, -0.003)
Public facility availability (count/10,000 population)						
Males	-0.037	(-0.077, 0.004)	0.006	(-0.073, 0.085)		
Females	0.053*	(0.008, 0.097)	-0.025	(-0.126, 0.076)		
Alpha Index	--^2^		--^2^		-0.006	(-0.101, 0.089)
Median household income						
Males	--^2^		--^2^		0.017	(-0.021, 0.055)
Females	--^2^		--^2^		-0.032	(-0.071, 0.007)
Crime						
Males	-0.099*	(-0.134, -0.065)	-0.135*	(-0.195, -0.075)	--^2^	
Females	-0.043*	(-0.081, -0.005)	-0.079*	(-0.142, -0.015)	--^2^	

^1^National Longitudinal Study of Adolescent Health (U.S.; n = 12,701)^. ^Estimated from Poisson fixed effects regression modeling MVPA as a function of six natural log-transformed built and socioeconomic environment measures. Fixed effects models adjusted for time varying age and do not estimate parameters for time invariant individual-level variables; random effects models additionally adjusted for sex, race, parental income and education, and region.

^2^Estimate for total sample reported if corresponding interaction with residential relocation was not included in the model. Residential relocation was defined as greater than 1/4 mile Euclidean distance (Mover (n = 8,525) and Non-mover (n = 4,176)) between Wave I and III respondent locations. 3- and 2-way interactions between sex, residential relocation status, and environment measures were included if statistically significant (p < 0.1); if a 3-way interaction was significant, all corresponding 2-way interactions were retained.

*Statistically significant elasticity (2-sided p < 0.05)

CI, Confidence Interval; MVPA, moderate-vigorous physical activity (bouts per week)

## Discussion

We estimated longitudinal effects of built and socioeconomic environment characteristics on MVPA bouts in a prospective study of adolescents as they transition into young adulthood. To our knowledge, ours is the first study to examine built environment changes resulting from either residential relocation or changes around stationary residents. After adjusting for unmeasured time invariant characteristics, MVPA bouts were higher with greater availability of pay facilities in males, and lower with higher crime in males and females. Other associations were null or in the counter-intuitive direction. However, we discuss several methodological considerations in the following sections.

### Built environment findings in the Add Health population

In contrast to relatively consistent cross-sectional associations between the built environment and PA in the extant literature [38,39], many cross-sectional [40] and random effects associations were weak or null in the Add Health population. Possible methodological explanations for these differences include our buffer-based environment measures and complications related to broad geographic variation and measurement of complex environments [20]. In another longitudinal, national study, urban sprawl was weakly related to obesity [8]; however, we expected a stronger, more robust relationship with PA, a more proximal outcome. Additionally, theorized behavior-specific relationships [41] such as promotion of walking for transit by highly connected streets could not be examined with our total leisure-time MVPA measure. Of course, null associations may reflect a lack of causal effects. Ultimately, several naïve estimates (cross-sectional and random effects) were null or counterintuitive, so corresponding within-person estimates cannot be attributed solely to adjustment for unmeasured time invariant characteristics.

### Residential self-selection bias: upward, downward, or more complex?

Residential self-selection is typically presented as a positive confounder which may create or magnify associations between the built environment and PA [5,6,42]. This characterization assumes that hypothesized built environment PA supports are: (1) preferred by or correlated with other neighborhood characteristics selected by people with higher PA (e.g., high performing schools), or (2) uncommon in areas selected by people with generally lower PA (e.g., lack of resources in affordable neighborhoods). These assumptions are supported by disproportionate allocation of recreation resources to more affluent neighborhoods [3,43-45] and by attenuation of relationships between urban form and health-related outcomes by first difference models [8] and other adjustment methods [5,46,47].

However, some PA-promoting features may be less common in advantaged areas. For example, pay facilities may encourage PA but may be more common in commercial centers potentially selected *less *often by advantaged families (with higher PA levels). In this scenario, residential self-selection factors are negative confounders, consistent with stronger positive estimated within-person (versus random) effects of pay facilities on MVPA in males.

In contrast, within-person (versus random effects) estimates of higher crime effects on lower MVPA were attenuated in females, suggesting that self-selection factors related to crime may operate differently in females versus males. That is, crime and safety may play a stronger role in not only MVPA but also selection of a neighborhood in females than in males. Overall, these results suggest that residential self-selection may magnify or attenuate built environment-PA associations and involves multifaceted relationships among complex environments and sex-specific determinants of residential selection and PA.

Furthermore, concerns that selection of neighborhoods based on activity-related amenities can explain positive environment-PA associations [5] suggests positive confounding but not necessarily absence of causal effects. That is, selected amenities may help active individuals to maintain or increase their activity levels, formally defined as "effect in the treated" [48]. Alternatively, "effect in the untreated" would support placement of activity-related amenities in areas of greatest need. Investigation of heterogeneous effects may clarify the potential value of various built environment modification strategies.

### Within-person estimators applied to a life transition period

Within-person estimators control for unmeasured characteristics that remain constant over time, a major strength for addressing residential selection factors, which are challenging, if not impossible, to measure accurately [6]. However, examination of neighborhood effects during the adolescence to young adulthood transition raises several complications:

#### Time varying characteristics

Within-person estimators do not control for unmeasured characteristics which change over time. Residential relocation is typically triggered by marriage, childbearing, employment opportunities [16], or other events which characterize the adolescent to young adulthood transition [49] and may lead to changes PA. Sedentary employment or intensive schooling in young adulthood may reduce PA levels, overwhelming any built environment effects on PA. Such events may also influence the type of neighborhood selected, thus comprising time varying, potentially unmeasured confounders.

Because these events are rare in adolescence, there was insufficient variability in Wave I for analysis as time varying measures. For example, magnification of negative crime-MVPA associations by within-person estimation in males could be explained by movement into urban centers (with higher crime) for employment, which may limit leisure time for PA. Employment may therefore be a time varying confounder which is unmeasured in our study.

Importantly, similar residential relocation triggers may occur throughout middle and later adulthood, with similar implications for bias if they are not sufficiently measured. Further, because residential self-selection may attenuate estimated relationships, null associations do not necessarily imply that bias has been fully addressed. Exploration and development of approaches for addressing time-varying characteristics that are unmeasured is clearly an important area for future work. Possible strategies include instrumental variables methods or other simultaneous equation strategies which model predictors of residential selection and neighborhood predictors of behavior or health in two or more stages [6].

#### Age-specific effects

Our longitudinal models assume constant causal effects between time points [10], a questionable assumption during periods of shifting PA determinants. However, differences in published cross-sectional associations between Wave I and III were not statistically significant [40]. Nevertheless, estimated causal effects in adolescents versus young adults should be further investigated using longitudinal data and innovative adjustment strategies.

#### Residential selection by parents

Residential location was likely determined by parents in Wave I but respondents in Wave III. Therefore, the source of unmeasured residential selection factors varied across waves and may contribute additional bias. However, previous neighborhood characteristics are the most powerful predictors of subsequent neighborhood characteristics [50,51], suggesting that key unmeasured characteristics may remain constant and carry across generations.

#### Summary

Within-person estimation has limitations but is particularly relevant for capturing short-term effects theorized for behavioral outcomes such as PA [10] and is overall a valuable approach for addressing residential self-selection bias.

### Restriction by residential relocation status: an additional source of bias?

Biases related to residential stability may be at least as strong as residential relocation: in the adolescent to young adulthood transition, individuals may remain in the parent's home for reasons (e.g., care for young children, unemployment, or attendance at a local college) associated with health behaviors (outcomes), and neighborhoods (exposures) change systematically (e.g., disadvantaged groups more often live in neighborhoods with less advantageous environment trajectories [51]). Thus, conditioning on residential relocation may induce selection bias.

Indeed, movers and non-movers differ with regard to individual characteristics in this and prior studies [52] and to estimated environment-MVPA associations. With the exception of public facilities, associations were weaker or equivalent in movers than non-movers, but these patterns could be reversed in adulthood when residential stability is the norm.

Differential associations could also reflect different sets of unmeasured factors that influence residential selection (in movers) versus changes in neighborhoods around stationary residents (non-movers). In the full sample, we expect residential selection factors to dominate because the majority of the sample moved between Waves I and III. However, distinguishing between selection bias and differential confounding is complex and requires future research using analytical methods such as marginal structural models that can address relocation status without inducing selection bias through covariate adjustment or stratification [17].

### Strengths and limitations

Limitations of this study include the methodological concerns raised above. Additionally, our definition of residential relocation did not capture duration of residence and may have misclassified respondents who moved short distances or moved but returned to the same location by Wave III. Second, changes in socioeconomic environment variables around a given location may reflect shifts in census boundaries between 1990 and 2000. Also, there was temporal mismatch between interview data and census and street connectivity data; in particular, temporal mismatch in Wave I was a tradeoff for greater accuracy of a more current street database. Third, neighborhood buffers delineated by street network distance may yield different results; however, population counts needed for our facility availability measures were not available within network buffer areas, and environment measures are similar for Euclidean versus network distance-based buffers. Additionally, conversion of population within buffers from population within block groups (Table 1) may have resulted in measurement error in our facilities availability measures and bias of unpredictable direction and magnitude in corresponding associations with MVPA, particularly in heterogenous areas. Fourth, our data sources may have captured relevant neighborhood characteristics more completely in some subgroups (e.g., our database does not capture PA resources on college campuses), potentially resulting in differential measurement error by study wave or sociodemographic group. Fifth, the PA environments at school, workplace, or other locations were not addressed in this study.

Loss to follow-up and missing individual-level data could have led to biased estimates. Our leisure time MVPA frequency measure does not distinguish between possible behavior-specific effects [41] (e.g. promotion of active transit versus exercise); incorporate physical activity duration or intensity; and may have systematically omitted important activities which could account for the observed sex differences. Also, while our Wave I MVPA measure was based on instruments validated in other epidemiologic child and adolescent studies, modifications made for Wave III (addition of age-appropriate activities) has not been validated in young adults. However, these are tradeoffs for the size and scope of the Add Health study. Finally, the direction of effect remains ambiguous, as we examined simultaneous changes in the environment and in MVPA bouts.

However, our unique time-varying environment database captures residential locations of a large, nationally representative population followed through a critical life stage. By including six built and socioeconomic environment measures shown to adequately represent key environmental constructs, we addressed environmental confounders while avoiding collinearity. Our longitudinal data was used to address residential self-selection bias and explore bias related to residential relocation.

## Conclusions

After controlling for residential self-selection bias using within-person estimators, MVPA bouts were related only to pay facility availability in males and crime in males and females in the expected directions. Our results suggest that the magnitude and direction of residential self-selection bias can vary across environmental and individual characteristics. Within-person estimators are valuable for controlling for residential self-selection bias, but their application to the adolescence to young adulthood transition or other major life transitions is complex. Further research and development of methods that can address predictors of residential relocation while simultaneously controlling for unobserved measures is needed.

## Competing interests

The authors declare that they have no competing interests.

## Authors' contributions

JB-H designed the study, performed statistical analyses, and drafted the manuscript. DKG participated in study design, provided statistical consultation, and critically reviewed the manuscript. KRE provided study design feedback and critically reviewed the manuscript. PG-L supervised all aspects of the study. All authors read and approved the manuscript.

## Supplementary Material

Additional file 1 **Appendix A, Unmeasured variables in fixed effects models**. Detailed description of fixed effects models and how they control for time constant unmeasured variables. Click here for file

Additional file 2 **Appendix B, Supplemental tables**. Model coefficients and p-values for main effects and interaction terms (Tables B1 and B2) corresponding to effect estimates reported in Tables 4 and 5. Click here for file
