# Supplementary material for: Residential self-selection bias in the estimation of built environment effects on physical activity between adolescence and young adulthood
Source: Int J Behav Nutr Phys Act. 2010 Oct 4;7:70. doi: 10.1186/1479-5868-7-70 (PMC2959083; doi:10.1186/1479-5868-7-70)
Supplement: Additional file 1 — Appendix A, Unmeasured variables in fixed effects models. Detailed description of fixed effects models and how they control for time constant unmeasured variables. [file 1479-5868-7-70-S1.DOC]

**APPENDIX A**

Unmeasured variables in fixed effects models

To illustrate how fixed effects models address unmeasured, time invariant variables, consider Model 1, which models PA as a function of a neighborhood environment characteristic of interest (E), vectors of observed time invariant and variant sociodemographic variables (S and T, respectively), and vectors of unobserved time invariant and variant variables (U and V, respectively). Associated error terms are ε_i_ (person-specific error) and ν_it_ (error for person i at time t), which are assumed to be normal. In random effects models, the intercept (β_0i_) is estimated for each individual.

(1) PA_it_ = β_0i_ + β_1_E_it_ + β_2_S_i_ + β_3_T_it_ + β_4_U_i_ + β_5_V_it_ + ε_i_ + ν_it_

Because U and V are unmeasured, the variability in PA explained by U and V is captured in composite errors ε_i_`=β_4_U + ε_i_ and ν_it_`=β_5_V + ν_it_, respectively. If ε` or ν_it_` is correlated with E, S, and T, standard estimation methods will lead to biased estimates of β_1_, β_2_ and β_3_. Given Model 1, the following model is also true [28], where * indicates the within-person average of time-varying variables over time:

(2) PA_it_* = β_0i_ + β_1_E_i_* + β_2_S_i_ + β_3_T_i_* + ε_i_` + ν_i_`*

Subtracting (2) from (1) yields the following model, which is estimated by fixed effects models:

(3) (PA_it_ - PA_i_*) = β_1_(E_it_ - E_i_*) + β_3_(T_ij_ - T_i_*) + (ν_it_` - ν_i_`*)

Time invariant sociodemographics (S) and person-specific error (ε_i_`) subtract out of Model 3. In particular, ε_i_` captures unmeasured time invariant factors, which will no longer bias the estimates. Interpretation of the coefficients is unchanged from traditional regression models. A more detailed description of a similar type of within-person estimator (first difference models) is provided in a prior publication [6].
