# Supplementary material for: Residential self-selection bias in the estimation of built environment effects on physical activity between adolescence and young adulthood
Source: Int J Behav Nutr Phys Act. 2010 Oct 4;7:70. doi: 10.1186/1479-5868-7-70 (PMC2959083; doi:10.1186/1479-5868-7-70)
Supplement: Additional file 2 — Appendix B, Supplemental tables. Model coefficients and p-values for main effects and interaction terms (Tables B1 and B2) corresponding to effect estimates reported in Tables 4 and 5. [file 1479-5868-7-70-S2.DOC]

**APPENDIX B**

Supplemental tables

**Table B1. Model coefficients and significance for random and within-person effect estimates^1^ of built and socioeconomic environment characteristics on MVPA between adolescence (Wave I, 1994-95) and young adulthood (Wave III, 2001-02)**

|  | Within-Person Effects | | Random Effects | |
| --- | --- | --- | --- | --- |
|  | Coefficient (95% CI) | *P* | Coefficient (95% CI) | *P* |
| Landscape diversity | -0.018 (-0.037, 0.001) | 0.07 | -0.008 (-0.024, 0.008) | 0.32 |
| Pay facilities (count/10k population) | 0.024 (0.006, 0.042) | 0.008 | 0.014 (0.000, 0.029) | 0.06 |
| Female*Pay facilities^2^ | -0.040 (-0.064, -0.015) | 0.001 | -0.026 (-0.045, -0.007) | 0.007 |
| Public facilities (count/10k population) | 0.002 (-0.025, 0.030) | 0.88 | 0.008 (-0.016, 0.032) | 0.49 |
| Alpha street connectivity | -0.002 (-0.097, 0.092) | 0.96 | -0.015 (-0.088, 0.058) | 0.69 |
| Median household income | 0.022 (-0.016, 0.060) | 0.25 | -0.019 (-0.048, 0.010) | 0.19 |
| Female*Median household income^2^ | -0.061 (-0.115, -0.007) | 0.03 | 0.033 (-0.003, 0.070) | 0.07 |
| Crime (per 100,000 population) | -0.107 (-0.140, -0.075) | <0.001 | -0.056 (-0.083, -0.029) | <0.001 |
| Female *Crime^2^ | 0.061 (0.013, 0.109) | 0.012 | -0.005 (-0.036, 0.025) | 0.74 |

CI, Confidence Interval; MVPA, moderate-vigorous physical activity (bouts per week)

^1^National Longitudinal Study of Adolescent Health (U.S.; n=12,701). Corresponds with estimates reported in Table 4. Estimated from Poisson random and fixed effects regression modeling MVPA as a function of natural log-transformed built and socioeconomic environment measures. Fixed effects models adjusted for time varying age and do not estimate parameters for time invariant individual-level variables; random effects models additionally adjusted for time invariant sex, race, parental income and education, and region.

^2^Sex interactions were included if significant (p<0.1) in either random or fixed effects models.

**Table B2. Model coefficients and significance for within-person effect estimates^1^ of built and socioeconomic environment characteristics on MVPA between adolescence (Wave I, 1994-95) and young adulthood (Wave III, 2001-02) by residential relocation status^2^**

|  | Coefficient (95% CI) | *P* |
| --- | --- | --- |
| Landscape diversity | -0.072 (-0.116, -0.029) | 0.001 |
| Mover*Landscape diversity | 0.068 (0.020, 0.116) | 0.006 |
| Pay facilities (count/10k population) | 0.027 (0.009, 0.045) | <0.001 |
| Female*Pay facilities | -0.050 (-0.075, -0.024) | <0.001 |
| Public facilities (count/10k population) | 0.006 (-0.073, 0.085) | 0.884 |
| Mover*Public facilities | -0.043 (-0.130, 0.045) | 0.344 |
| Female*Public facilities | -0.031 (-0.158, 0.097) | 0.64 |
| Mover*Female*Public facilities | 0.120 (-0.020, 0.259) | 0.09 |
| Alpha street connectivity | -0.006 (-0.101, 0.089) | 0.90 |
| Median household income | 0.017 (-0.021, 0.055) | 0.38 |
| Female*Median household income | -0.050 (-0.104, 0.005) | 0.08 |
| Crime (per 100,000 population) | -0.135 (-0.195, -0.075) | <0.001 |
| Mover*Crime | 0.036 (-0.025, 0.097) | 0.25 |
| Female*Crime | 0.057 (0.009, 0.104) | 0.02 |

CI, Confidence Interval; MVPA, moderate-vigorous physical activity (bouts per week)

^a^ National Longitudinal Study of Adolescent Health (U.S.; n=12,701). Corresponds with estimates reported in Table 5. Estimated from Poisson fixed effects regression modeling MVPA as a function of natural log-transformed built and socioeconomic environment measures. Fixed effects models adjusted for time varying age and do not estimate parameters for time invariant individual-level variables.

^b^Residential relocation was defined as greater than (mover) versus less than ¼ mile Euclidean distance between Wave I and III respondent locations. 3- and 2-way interactions between female sex, residential relocation, and environment measures were included if statistically significant (p<0.1); if a 3-way interaction was significant, all corresponding 2-way interactions were retained.
